# Research Progress on the Structure and Function, Immune Escape Mechanism, Antiviral Drug Development Methods, and Clinical Use of SARS-CoV-2 M^pro^

**DOI:** 10.3390/molecules30020351

**Published:** 2025-01-16

**Authors:** Jiayi Ren, Zhengfu Zhang, Yi Xia, Daqun Zhao, Dingqin Li, Shujun Zhang

**Affiliations:** Department of Biochemistry and Molecular Biology, School of Basic Medical Sciences, Southwest Medical University, Luzhou 646000, China; rjy8891@163.com (J.R.); 20200140330135@stu.swmu.edu.cn (Z.Z.); xiayi20020524@163.com (Y.X.); zhaodaqun168@swmu.edu.cn (D.Z.)

**Keywords:** SARS-CoV-2, main protease (M^pro^), structure and function, immune escape, drug development methods, antiviral drug

## Abstract

The three-year COVID-19 pandemic ‘has’ caused a wide range of medical, social, political, and financial implications. Since the end of 2020, various mutations and variations in SARS-CoV-2 strains, along with the immune escape phenomenon, have emerged. There is an urgent need to identify a relatively stable target for the development of universal vaccines and drugs that can effectively combat both SARS-CoV-2 strains and their mutants. Currently, the main focus in treating SARS-CoV-2 lies in disrupting the virus’s life cycle. The main protease (M^pro^) is closely associated with virus replication and maturation and plays a crucial role in the early stages of infection. Consequently, it has become an important target for the development of SARS-CoV-2-specific drugs. This review summarizes the recent research progress on the novel coronavirus’s main proteases, including the pivotal role of M^pro^ in the virus’s life cycle, the structure and catalytic mechanism of M^pro^, the self-maturation mechanism of M^pro^, the role of M^pro^ in virus immune escape, the current methods of developing antiviral drugs targeting M^pro^, and the key drugs that have successfully entered clinical trials. The aim is to provide researchers involved in the development of antiviral drugs targeting M^pro^ with systematic and comprehensive information.

## 1. Introduction

**Figure 1:**
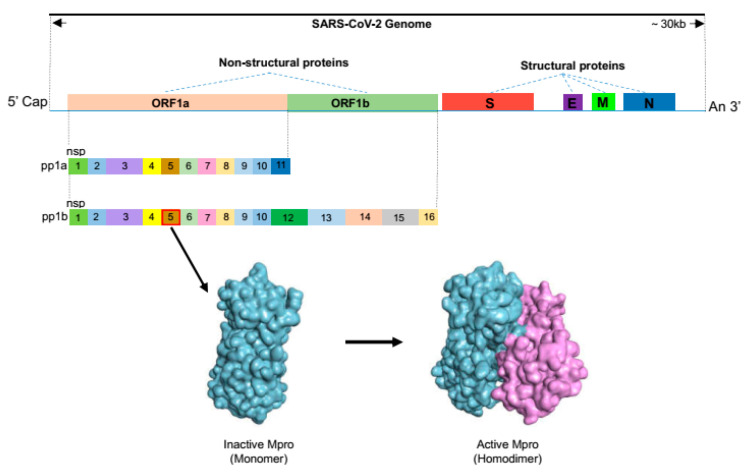
Genome organization of SARS-CoV-2 and structural overview of the SARS-CoV-2 main protease.

SARS-CoV-2 belongs to the Coronaviridae family and is a positive-strand RNA virus with a genome of approximately 30,000 bases (Figure 1) [1,2,3]. It contains two large overlapping open reading frames (ORF1a and ORF1b) and encodes four structural proteins, i.e., spike, envelope, membrane, nucleocapsid (N) proteins, and nine accessory proteins [2]. Among these, the outermost spike protein (S protein) primarily functions in binding to the angiotensin-converting enzyme 2 (ACE2) on the surface of human cells, driving the coronavirus to infect human cells [4]. Notably, this region is highly susceptible to mutations [5,6,7]. The envelope (E) protein is necessary for viral pathogenesis, and its smaller size facilitates easier viral assembly before release. Meanwhile, the membrane (M) protein plays a central role in viral assembly, making cellular membranes into workshops, where viruses and host factors come together to create new virus particles [8]. The innermost nucleocapsid protein spans 419 amino acids and encompasses three intrinsically disordered regions (IDRs) and two conserved structural regions (CSRs) [9]. The IDRs consist of an N-terminal disordered structure (N-arm), a central Ser/Arg-rich flexible linker region (LKR) located midway in the protein sequence, and a C-terminal disordered structure (C-tail). The CSRs include an N-terminal domain (NTD) and a C-terminal domain (CTD) [9,10]. Among these regions, the NTD is responsible for RNA binding; the CTD is responsible for both RNA binding and dimerization, and the IDRs regulate RNA binding activity as well as the oligomerization of NTD and CTD [10,11,12,13]. ORF1a and ORF1b are further processed by the M^pro^ and catalase to produce 16 non-structural proteins (Nsp1 to 16) [14]. These non-structural proteins play various roles throughout viral replication and maturation [2,14,15]. The essential role of M^pro^ in viral replication and maturation makes it an ideal and unique target for antiviral therapy [16]. Here, we summarize the drug development methods targeting SARS-CoV-2 M^pro^ and provide an overview of the current primary clinical drugs for the treatment of COVID-19.

## 2. The Role of SARS-CoV-2 M^pro^ in the Life Cycle

**Figure 2:**
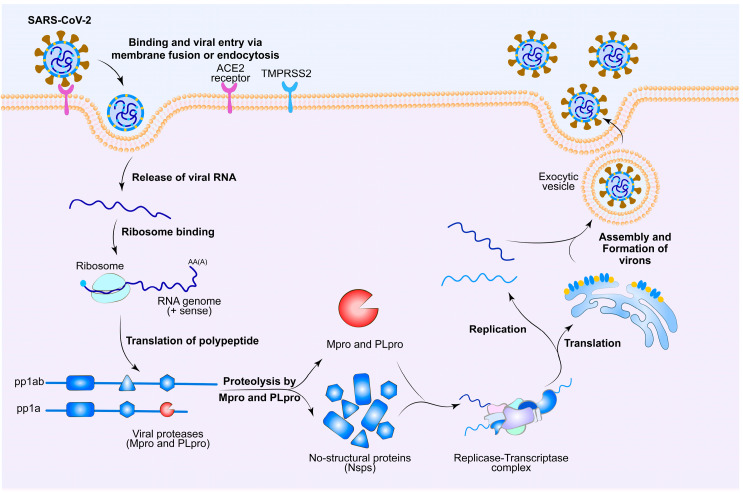
The life cycle of the SARS-CoV-2 inside the host cell. Created with BioRender.com.

The life cycle of SARS-CoV-2 is depicted in Figure 2. The spike (S) protein located on the surface of SARS-CoV-2 specifically binds to the ACE2 receptor on the surface of host cells [14,17,18,19,20,21,22,23,24]. Subsequently, the transmembrane protease serine 2 (TMPRSS2) of the host cell facilitates the cleavage of the S protein. This exposed site promotes the fusion of the viral envelope with the host cell membrane, allowing the virus to enter the host cell. Upon entry, the N protein facilitates the release of viral RNA through capsid dissociation. The N protein is dissociated from the positive-sense (+) RNA genome of the virus, initiating a tightly regulated viral genome replication and expression program in both space and time [13,24]. On the one hand, host cellular mechanisms convert the genetic information into viral proteins required for replication. The positive-sense (+) RNA genome of the virus translates ORF1a and ORF1b into overlapping polyproteins pp1a and pp1ab, respectively (Figure 1) [24,25]. Subsequently, these polyproteins are then cleaved into 16 mature, non-structural proteins (NSPs) by M^pro^ and papain-like protease (PLpro) [26,27,28]. M^pro^, formed by NSP5, cleaves these two polyproteins at 11 recognition sites to generate NSP4 to NSP10 and NSP12 to NSP16 (NSP11 being the N-terminus of NSP12), while PLpro cleaves at three additional sites to produce NSP1 to NSP3 [29,30,31,32]. The NSPs cleaved by M^pro^, from NSP4 to NSP16, contain many essential viral proteins, particularly the RNA-dependent RNA polymerase (NSP12), RNA-binding protein (NSP9), helicase (NSP13), ribonuclease exonuclease (NSP14), and methyltransferase (NSP16) [27,33,34]. Therefore, effective blockade of M^pro^ can prevent SARS-CoV-2 replication in humans and cure the disease. On the other hand, the non-structural proteins (nsp2–16) produced by the hydrolysis of M^pro^ and PLpro assemble into the viral replicase–transcriptase complex. This complex uses the positive-sense (+) RNA as a template to synthesize negative-sense (−) RNA and subgenomic (−) RNA through a discontinuous extension mechanism. The previously synthesized RNA serves as a template for generating new positive-sense RNA, which encodes structural proteins. Simultaneously, the virus induces alterations in the endoplasmic reticulum (ER), transforming it into vesicular structures known as double-membrane vesicles (DMVs), which aid in the replication and translation of viral RNA [24,25]. Subsequently, the S, E, and M proteins are transported to the ER, and the N protein binds to positive-sense RNA to form nucleoprotein complexes, completing the assembly of virions in the Golgi apparatus [24]. Before virion release, the host cell’s Flynn protease cleaves five amino acids at critical sites of the S protein, generating infectious virons [13,14,15,24]. These virons ultimately release from infected host cells, completing their life cycle [24,25]. It is evident from this that M^pro^ plays a crucial role in the life cycle of SARS-CoV-2, making it a highly attractive drug target for the development of antiviral drugs against COVID-19 [35].

### 2.1. The Structure and Function of M^pro^

**Figure 3:**
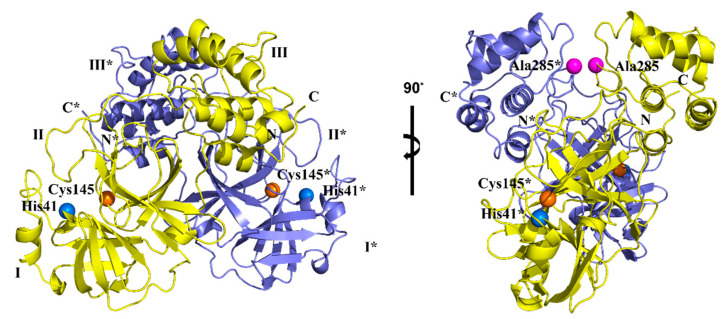
The 3D structure of SARS-CoV-2 M^pro^ in two different views. One protomer of the dimer is shown in light blue and marked with “*” accordingly, the other one in yellow.

The crystal structure of the first SARS-CoV-2 M^pro^ was determined using X-ray diffraction with a resolution of 2.16 Å and deposited in the protein databank (PDB) by Jin et al. [36], published on 5 February 2020 (PDB ID:6LU7). To date, more than 1000 M^pro^ structures have been stored in PDB, providing unprecedented insights into the study of M^pro^ structure and catalytic mechanism [37]. The M^pro^ enzyme has a molecular weight of 33.8 kDa and exists as a homodimer (Figure 3) [16].

Its structure consists of two protomers (A and B). Upon dimerization and activation, M^pro^ adopts a proper conformation to carry out its catalytic function [38,39,40,41]. The two protomers are arranged linearly with each other [16,42]. Each protomer comprises 306 amino acid residues and contains three unique functional domains: Domain I (residues 8–101); Domain II (residues 102–184); and Domain III [43]. Domains I and II are characterized by antiparallel β-barrels. The cleft between Domains I and II is lined with hydrophobic residues, within which the substrate-binding site and the catalytic site are located [16,39,44,45]. Domain III consists of two antiparallel globular clusters composed of five α-helices each, responsible for catalytic activity [16,39]. Domain III is connected to Domain II through two extended annular regions [36,42]. In the SARS-CoV M^pro^ structure, there is a hydrogen bond between Thr285 residues from the two domains’ III units. This interaction is consistent with the sparse interaction between Thr285 and Ile286, maintaining the original conformation of SARS-CoV M^pro^ [39]. However, in SARS-CoV-2 M^pro^, the corresponding residues Thr and Ile are replaced by Ala and Leu, respectively, which brings the two domains’ three units closer together [36,46]. As a result, the catalytic activity of the enzyme is increased by 3.6 times, and the catalytic turnover rate is also enhanced [47,48]. This may be one of the reasons why SARS-CoV-2 is more infectious than its predecessors [39].

**Figure 4:**
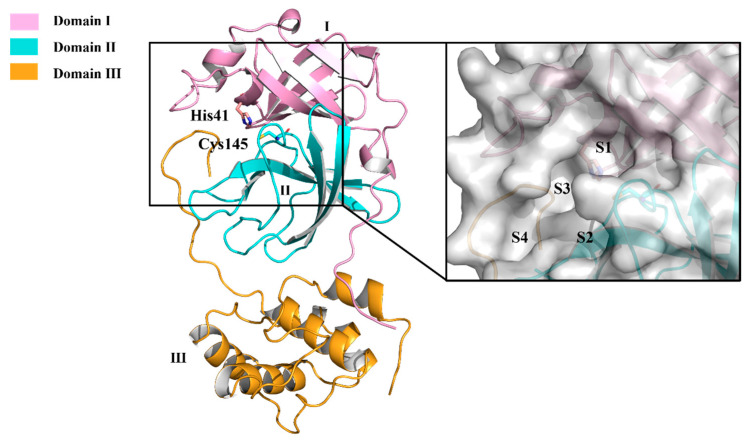
Crystal structure of free SARS-CoV-2 M^pro^ (**left**) and surface view of the substrate-binding cleft (**right**). The three distinct domains of the protomer are indicated. His41 and Cys145 residues of the catalytic dyad of Domain III are represented. The four subsites of the substrate-binding cleft are indicated (S1, S2, S3 and S4).

**Figure 5:**
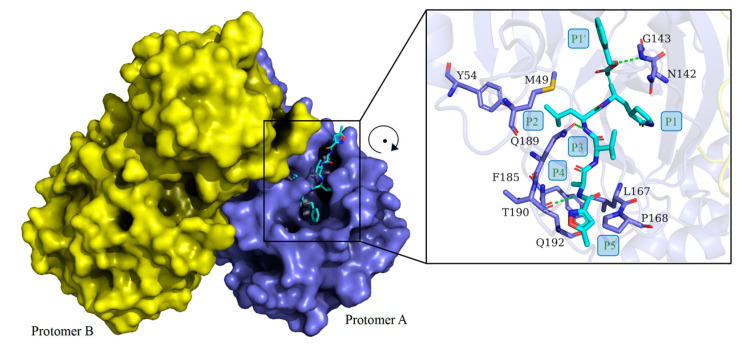
Structure of SARS-CoV-2 M^pro^ bound with inhibitors. **Left**: Surface representation of SARS-CoV-2 M^pro^ (PDB: 6LU7) with monomers in yellow and blue. Inhibitor is marked in cyan. **Right**: Close-up of the active site residues and interactions with the inhibitor N3.

Understanding the primary mechanism of peptide cleavage catalyzed by cysteine protease is crucial for designing effective structure-based inhibitors [49]. In the gap between domains I and II of M^pro^, two classical Cys–His pairs serve as catalytic sites, while other surrounding residues contribute to substrate specificity [50]. The cysteine residue of the Cys–His element structure initiates the nucleophilic attack on the substrate’s active site, while the histidine residue helps stabilize the intermediate state. In the vicinity of this complex, M^pro^ forms a conserved binding pocket consisting of four sites (S1, S2, S3, and S4) that can accommodate the substrate effectively (Figure 4) [49,51]. The amino acid residues of the substrate are typically numbered from the N-terminus to the C-terminus around the cleavage site as (P5-P4-P3-P2-P1↓P1’) (Figure 5). The typical sequence recognized and cleaved by M^pro^ within the polyprotein is Leu-Gln↓(Ser, Ala, Gly). Remarkably, a Gln residue and a long-side-chain hydrophobic residue are almost always required at the P1 and P2 positions, respectively [49,50].

**Figure 6:**
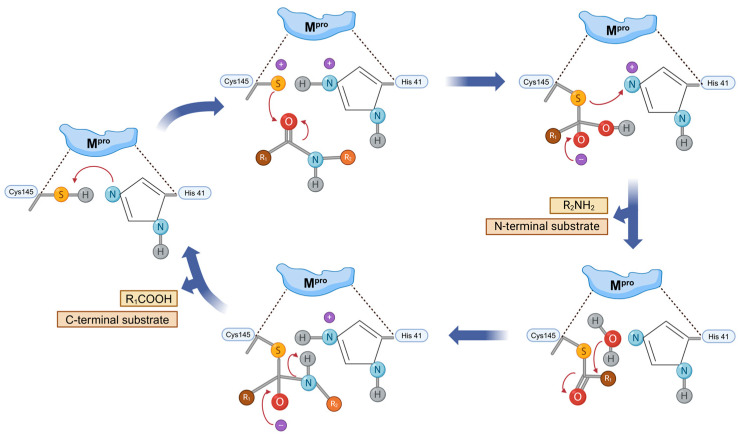
Hydrolysis mechanism of SARS-CoV-2 M^pro^. Amino acids of the catalytic dyad and the substrate are depicted in blue and red, respectively.

The catalytic mechanism of M^pro^ commences with the thiol deprotonation of Cys145 residue (Figure 6) [39,49,52]. His41 undergoes deprotonation of the—SH group of Cys145, resulting in the formation of the activated thiolate; this thiolate then performs a nucleophilic attack on the substrate’s carbonyl group, forming a tetrameric adduct. Subsequently, the product of peptide hydrolysis with an N-terminus segment is released, followed by the catalytic action of His41. The final step involves thioester cleavage, leading to the remaining peptide segment with the C-terminal [29,39,53].

### 2.2. Self-Assembly and Maturation Mechanism of M^pro^

Although the activity of M^pro^ is crucial for viral physiology, little is known about its maturation process. Throughout maturation processes, significant conformational changes occur in the active sites and surfaces of M^pro^, providing valuable insights for the development of targeted drugs. In 2005, Hsu’s study demonstrated that M^pro^ may form a small number of active dimers after self-cleavage, enabling the catalytic sites to act on other cleavage sites in polyproteins immediately [54]. In 2010, Li et al. [55] observed that the dimerization of mature M^pro^ is enhanced in the presence of substrates. They proposed that after translation, two M^pro^ propolymers transiently form a dimer stabilized by binding to the N-terminal site of its substrate (another M^pro^ in the polyprotein), which then undergoes further cleavage to release its N-terminal portion [55]. Additionally, Yang et al. [56] suggested that N-terminal autolysis may only require two immature forms of M^pro^ in monomer polyproteins to form intermediate dimers independently of the active dimer of mature enzymes.

Recently, Noske et al. [57] combined X-ray crystallography and chemical technology to provide further insights into the maturation process of SARS-CoV-2 M^pro^. After production within the Nsp5 domain of viral polymers, the immature form of M^pro^ contains N-terminal and C-terminal inserts, which require self-processing to generate mature enzymes. In M^pro^, the N-terminal cleavage site is situated between the two primers of the polymerase, serving as both parts of the polymerase boundary and part of the active site of each domain. In immature M^pro^, the additional amino acids at the N-terminal appear to disrupt the conformation of the active site at the S1–S3 site, thereby affecting its substrates’ recognition and processing abilities. Additionally, it also influences the ability of the enzymes to form dimers. By monitoring the time of dimer formation, Noske et al. [57] observed that the monomeric enzyme could cleave its N-terminal region, indicating that cis-cleavage was the first step in the maturation process. Furthermore, it was noted that the occurrence of dimer formation was much more frequent than N-terminal cleavage, suggesting the formation of transient dimers between two M^pro^s molecules, which stabilized through binding to the N-terminus of its substrate. This finding aligns with the model proposed by Li et al. [55] and Noske et al. [57], where partially cleaved M^pro^ leads to conformational constraints and reduced activity.

Moreover, the addition of C145S M^pro^ to normal M^pro^ appeared to significantly enhance N-terminal cleavage and dimer formation, indicating that this initial maturation step involves a combination of cis-and trans-cleavage events. Eventually, the N-terminal region is fully cleaved and packed within the polymer [57]. Once the active site region, including the C-terminal, undergoes maturation, the C-end of the M^pro^ polymer adopts an unusual rotational position, enabling its binding to the active site of another mature or semi-mature M^pro^ polymer. In this step, the trans-cleavage of the C-terminal residue acts as an anchor for the short-term polymer-polymer binding state of the protein. Subsequently, fully mature M^pro^ is produced, which then processes other segments of the viral polymer.

## 3. The Role of M^pro^ in SARS-CoV-2’s Immune Escape

The IFN/ISG system is an important early defense mechanism against viral infections, which plays a key role in protecting hosts from invasive pathogens [58,59,60]. Coronaviruses, including SARS-CoV-2, must overcome the IFN/ISG system in order to establish effective viral replication [58,61]. Type I IFNs (such as IFNα/β) are highly pleiotropic cytokines capable of inducing an antiviral state in infected cells and their neighboring cells by stimulating the production of cellular antiviral proteins collectively known as interferon-stimulated gene (ISG) proteins [62,63]. Through direct or indirect interactions with IFNs, ISGs achieve cellular effects such as antiviral defense and stimulation of adaptive immunity [64,65,66]. Upon binding to the cellular surface IFN receptor, secreted IFNs activate receptor-associated Janus kinases (JAK1 and TYK2), which, in turn, facilitate the recruitment and phosphorylation of signal transducer and activator of transcription proteins (STAT1 and STAT2). Phosphorylated STATs form dimers before interacting with IFN regulatory factor 9 (IRF9), collectively assembling into a heterotrimeric complex called interferon-stimulated gene factor 3 (ISGF3). The ISGF3 complex subsequently enters the nucleus to recognize IFN-stimulated response element (ISRE) sequences, leading to the expression of ISGs with antiviral functions [67]. However, studies have shown that severe COVID-19 patients often exhibit immune response dysregulation, including an uncontrolled pro-inflammatory response and impaired interferon response [58,61,68,69,70]. This indicates that SARS-CoV-2 has developed a mechanism to interfere with the production and function of IFN and ISG. Interestingly, the inhibition of M^pro^-mediated IFN reactions has been widely investigated for different vertebrate coronaviruses. For example, porcine triangular coronavirus (PDCoV) Nsp5 protease cleaves a regulatory protein called nuclear factor-κ B, which is necessary for IFN synthesis, thereby inhibiting the production of IFN [71]. Similarly, the SARS-CoV-2 M^pro^ has been reported to block the nuclear translocation of phosphorylated IRF3, leading to the inhibition of IFN induction [72]. Recently, Song et al. [60] have demonstrated that SARS-CoV-2 evades the antiviral immunity mediated by interferon by manipulating the activation of ISG. They found that the major protease of SARS-CoV-2 significantly inhibited the expression and transcription of downstream ISGs driven by interferon-stimulated response elements in a dose-dependent manner [60]. The overexpression of M^pro^ stimulated by IFN has been found to have significant effects on the transcription of various ISGs, including MX2, REC8 meiosis recombinant protein, OASL, mitogen-activated protein kinase, kinase 14, and IRF9 [73,74,75]. M^pro^ exerts its inhibitory effects on ISG production by cleaving histone deacetylase (HDAC) instead of directly targeting interferon signal transduction [60]. Upon host infection, SARS-CoV-2 utilizes M^pro^ to cleave HDAC at specific residues, such as Q261 and Q383, leading to the fragmentation of HDAC [60]. This results in the isolation of inactive HDAC fragments outside of the cell nucleus. Similar fragmentation events have been observed on HDAC1 and HDAC3, which are homologous members of HDACs [76,77,78]. The cleavage of HDACs by M^pro^ severely impairs their function. Furthermore, M^pro^ also deactivates the ISG effector mRNA decapping enzyme 1a (DCP1a) by cleaving its activity at the Q343 residue [60,79,80]. Although M^pro^ from AlphaCoronaVirus has weak catalytic activity against HDAC2, M^pro^ from different coronaviruses exhibits protease activity that can simultaneously cleave HDAC2 and DCP1a [80,81]. These findings highlight the role of SARS-CoV-2 M^pro^ as a key anti-immune effector that regulates the IFN/ISG system at multiple levels, aiding the virus in evading the host immune evasion.

In addition, Liu et al. [82] studied the mechanism of SARS-CoV-2 M^pro^ targeting RIG-I and MAVS to evade the innate immune response. M^pro^ cleaves 10 N-terminal amino acids from RIG-I, depriving it of its ability to activate MAVS. Additionally, M^pro^ promotes the ubiquitination and protein-mediated degradation of MAVS. By targeting RIG-I and MAVS, M^pro^ effectively inhibits the IFN response triggered by double-stranded RNA (dsRNA) in an enzyme-dependent manner. This study also demonstrated that small molecule inhibitors synthesized against M^pro^ could mitigate the damage caused by M^pro^ to cellular RIG-I and MAVS, as well as the processing of SARS-CoV-2 non-structural proteins. This restoration of the innate immune response hinders the replication of SARSCoV-2 [82]. These findings have a significant implication for the development of drugs targeting M^pro^ as a potential strategy for preventing SARS-CoV-2 infection and treating COVID-19.

## 4. Design and Synthesis of New Inhibitors

**Figure 7:**
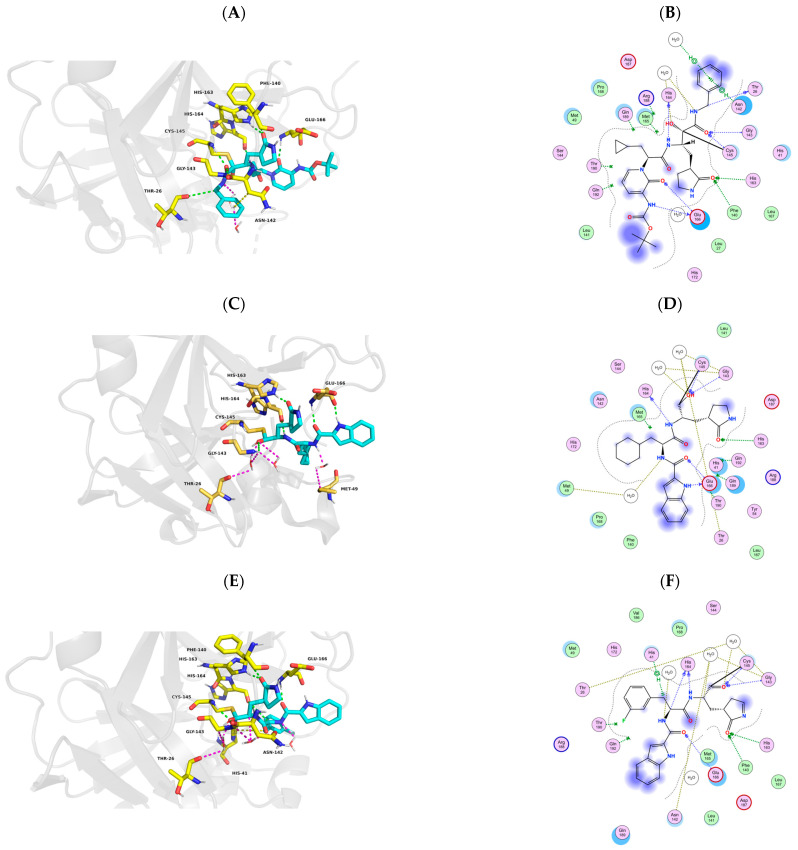

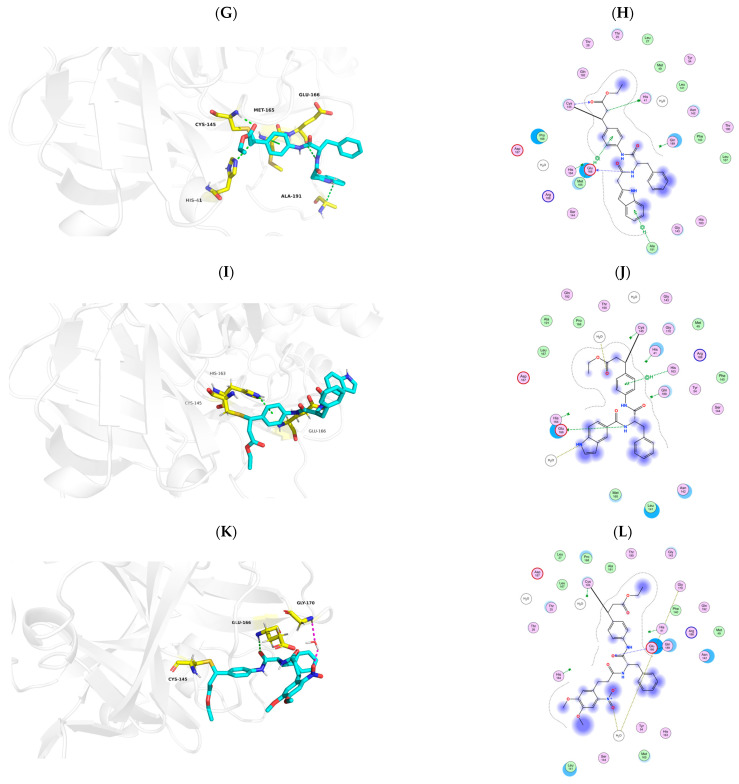
M^pro^-inhibitor binding modes. (**A**) Cartoon representation of the structure of SARS-CoV-2 M^pro^ in complex with compound 13b. (**B**) Schematic diagram of SARS-CoV-2 M^pro^-13b interactions. (**C**) Cartoon representation of the structure of SARS-CoV-2 M^pro^ in complex with compound 11a. (**D**) Schematic diagram of SARS-CoV-2 M^pro^-11a interactions. (**E**) Cartoon representation of the structure of SARS-CoV-2 M^pro^ in complex with compound 11b. (**F**) Schematic diagram of SARS-CoV-2 M^pro^-11b interactions. (**G**) Cartoon representation of the structure of SARS-CoV-2 M^pro^ in complex with compound 17. (**H**) Schematic diagram of SARS-CoV-2 M^pro^-17 interactions. (**I**) Cartoon representation of the structure of SARS-CoV-2 M^pro^ in complex with compound 18. (**J**) Schematic diagram of SARS-CoV-2 M^pro^-18 interactions. (**K**) Cartoon representation of the structure of SARS-CoV-2 M^pro^ in complex with compound 12. (**L**) Schematic diagram of SARS-CoV-2 M^pro^-12 interactions. All compounds are shown as light blue sticks.

So far, the structure and mechanism of M^pro^ binding to substrates have been studied relatively comprehensively, providing a solid basis for the design and synthesis of new M^pro^-targeting drugs [20,22,23,28,83,84,85,86,87]. Typically, newly designed drugs exhibit significant improvements in properties, such as metabolic stability, pharmacokinetic properties, and enhanced efficacy. Therefore, designing and synthesizing new drugs based on structure and the catalytic mechanism is one of the most common strategies in the search for anti-COVID-19 drugs (Figure 7) [25,88,89,90]. Using the structure-based method approach, Zhang et al. [46] proposed a novel inhibitor called 13b (Figure 7A,B). This newly developed compound demonstrated inhibition of SARS-CoV-2 M^pro^-suppressed viral RNA replication and exhibited enhanced inhibitory effects. Furthermore, it showed a longer half-life in blood plasma, with a clear propensity for pulmonary distribution, making it suitable for inhalation administration [91,92]. These favorable characteristics position 13b as a potential therapeutic agent against COVID-19. Yang et al. [93] designed a series of reversible covalent inhibitors based on b-(S-2-oxopyrrolidin-3-yl)-alanine, including dipeptidyl and tripeptidyl compounds. In vitro investigations in VeroE6 cells demonstrated excellent inhibition of M^pro^ by these compounds [84,93]. Choudhury et al. initially analyzed the binding of 191,678 molecular fragments to different constituent subsites of SARS-CoV-2 M^pro^. Subsequently, they selected fragments with high affinity to adjacent subsites and customized them to create new molecules. Through in silico computations, seventeen of these molecules exhibited promising binding capabilities [94]. Dai et al. [34] and Ramajayam et al. [95] proposed peptidomimetic aldehydes as potential antiviral drug candidates for structure-based ab initio drug design. They synthesized two compounds, 11a and 11b, utilizing peptidomimetic aldehydes as frameworks to covalently bind catalytic cysteines via their aldehyde groups, thereby inhibiting the activity of M^pro^ (Figure 7C–F). Additionally, these compounds showed favorable pharmacokinetic properties and low toxicity in animal models, warranting further investigation [34,95]. A series of M^pro^ inhibitors designed and optimized by Rathnayake et al. [96] were effective against a variety of human CoVs, including MERS-CoV, SARS-CoV, and SARS-CoV-2. Recently, Citarella et al. [97] designed novel inhibitors of SARS-CoV-2 M^pro^ using cinnamic ester. In this study, the researchers present a panel of *p*-aminocinnamic ethyl esters derivatives, which were joined to L-Phe residue variously decorated at the N-terminus with carbamate, urea, and indole-bearing amide recognition functionalities. The results indicated that the best inhibitor was the carbamate derivative 11, exhibiting a single-digit micromolar value (IC_50_ = 1.9 µM) [97]. Subsequently, all cinnamate derivatives were also tested against two representative human coronaviruses, hCoV-229E and hCoV-OC43. The findings revealed that indole-based amide inhibitors 17 and 18 effectively reduced the replication of hCoV-OC43 in the low micromolar range (EC_50_ of 9.14 µM and 10.1 µM, respectively), while the carbamate derivative 12 emerged as a potent and selective inhibitor of hCoV-229E replication (EC_50_ = 5.27 µM, Figure 7G–L) [97]. These results suggest that designing M^pro^ inhibitors based on the cinnamic ester for the treatment of human coronavirus infections is highly effective and feasible.

### 4.1. Drug Development Methods Targeting M^pro^

In the past two and a half years, the novel coronavirus has continued to evolve. Omicron variants have further mutated into the BA.1, BA.2, and BA.3.1 variants [98]. The Omicron variant BA.2 has emerged as the predominant strain in many regions [99]. The BA.1 variant has been found to exhibit significant evasion from neutralizing antibodies induced by vaccination [92,100,101,102]. A recent study estimated that BA.2 was approximately 1.5 times more infectious than BA.1 and demonstrated a better ability to evade 30% of the currently available vaccines compared to BA.1 [99]. Consequently, there is an urgent need to develop broad-spectrum antiviral drugs that are effective against these variants. M^pro^ plays a crucial role in the maturation of functional polypeptides that are involved in the assembly of the replication–transcription machinery [50,103].

### 4.2. Screening of Drugs Agafigureinst M^pro^ by Computational Methods

After the outbreak of COVID-19, the crystal structure of SARS-CoV-2 M^pro^ was rapidly determined, which greatly facilitated mechanistic research and the development of inhibitors [36,50,84,92,104]. Computational methods are commonly employed to identify potential therapeutic agents. These methods include structure-based virtual screening [105,106,107], ligand-based virtual screening [36,108], molecular docking [109,110], and molecular dynamics simulation [16,111]. These techniques enable the calculation-based identification of drug-like or lead-like candidates for targeting SARS-CoV-2 M^Pro^. For instance, Fischer et al. conducted a computational search for M^pro^ inhibitors by employing shape screening, smina docking, Glide docking, clustering, pharmacokinetic descriptors, MD simulations, and toxicity assessment. They extracted 13 hits from 606 million compounds in the ZINC database [112]. Ton et al. [113] utilized the deep docking method in conjugation with Glide to evaluate the inhibitory effect of 1.3 billion compounds in the ZINC15 library. They successfully identified 1000 potential compounds that exhibited excellent docking results at the active site of SARS-CoV-2 M^pro^ [113]. Joshi et al. [114] employed a multi-target-directed approach to discover ligands for M^pro^. Among approximately 7100 molecules, they identified nine natural compounds that displayed potent binding not only to M^pro^ but also to other targets such as ACE2 and RdRp [114]. The ability of these inhibitors to simultaneously target multiple essential proteins suggests their potential as antiviral agents, warranting further investigation [114]. Furthermore, numerous reports have emerged regarding the virtual screening of drug candidates targeting M^pro^, leading to the discovery of thousands of potential therapeutic drugs [106,115,116,117,118,119]. These promising compounds have been classified based on their chemical structure or biological activity, encompassing categories such as ketones, peptides, terpenes, quinolines, nucleoside and nucleotide analogs, protease inhibitors, phenolics, and sodium derivatives [50]. These research endeavors have significantly expedited the identification of suitable drug candidates against COVID-19.

### 4.3. Drug Repurposing

The development of a new, selective, and safe drug for the treatment of COVID-19 would be the most effective approach against this new epidemic disease. However, this is a time-consuming method and not in line with the current emergency. Therefore, the World Health Organization made a rational decision in early 2020 to prioritize the availability of existing antiviral drugs [39]. Based on the inhibition of SARS-CoV, MERS-CoV, hepatitis C, and other viruses, drug repurposing rapidly emerged as an antiviral strategy for SARS-CoV-2 [37]. Jin et al. [36] reported a novel repurposed drug for M^pro^ based on the N3 inhibition mechanism. N3 is a peptide-like inhibitor designed initially to treat infectious diseases caused by other CoVs [120,121,122,123]. It was subsequently observed that N3 also exhibited potent inhibition of the M^pro^ of SARS-CoV-2 [36,120]. The inhibition mechanism was elucidated by determining the crystal structure of the complex formed by SARS-CoV-2 M^pro^ and N3 [36]. The recognition mechanism between N3 and M^pro^ represents a typical pattern of inhibitor binding to M^pro^, as the substituents of N3 span all substrate binding subsites. Based on this mechanism, Jin et al. [36] screened over 10,000 compounds from a library of approved drugs, drug candidates in clinical trials, and other pharmacologically active compounds. They identified effective SARS-CoV-2 inhibitors such as disulfiram, carmofur, Ebselen, shikonin, tideglusib, PX-12, and TDZD-8. In another study, Günther et al. [124] selected 5953 compounds from two libraries for combined crystallization, leading to the identification of 29 distinct binders based on crystallographic screening of M^pro^. Furthermore, antiviral activity evaluation of these compounds can be achieved through cell-based experiments against SARS-CoV-2. Surprisingly, nine compounds reduced viral RNA replication by 100-fold. Among the most effective compounds were Calpeptin and Pelitinib, which are promising candidates suitable for preclinical testing and further investigation [124].

### 4.4. Experimental Screening

Ma et al. [125] identified drug candidates for M^pro^ through experimental screening. Using the FRET-based enzymatic detection method, they discovered that prednisone, boceprevir, GC-376, and calmodulin inhibitors II and XII exhibited strong inhibitory effects on M^pro^. In the in vitro antiviral activity tests, these repurposed drugs also demonstrated potent inhibition of SARS-CoV-2 replication in cell culture [125]. Su et al. [126] found that components of traditional Chinese medicine could also inhibit SARS-CoV-2 by targeting M^pro^. Baicalin and baicalein are natural products derived from *Scutellaria baicalensis*, which are often used in the prevention and treatment of hepatitis and respiratory diseases [127] and showed significant inhibition of M^pro^ in this study. The structure of the M^pro^-baicalein complex revealed a novel covalent inhibition model. Due to its unique antiviral activity in vitro and favorable safety profile in clinical trials, baicalin has emerged as a promising candidate for clinical trials and a potential lead compound for M^pro^ inhibition [127].

## 5. Clinical Drugs with M^pro^ Inhibitory Effect

The U.S. Food and Drug Administration (FDA) officially approved Pfizer’s new coronavirus infection treatment drug, Paxlovid, which was granted emergency use authorization by the FDA in December 2021 FDA [128]. The drug is specifically recommended for individuals over the age of 50 and those infected with the new coronavirus and having underlying diseases. Paxlovid is composed of Nirmatrelvir, an M^pro^ inhibitor, co-packaged with ritonavir. Ritonavir, although not the active ingredient that binds directly to M^pro^, functions as a modulator that prolongs the duration of nimatclovir and increases drug plasma concentrations to inhibit SARS-CoV-2 replication [102,129]. Before Paxlovid, PF-00835231 and its phosphate original drug PF-07304814 were the first anti-M^pro^ compounds to enter clinical trials (Table 1) [130]. PF-00835231 has undergone in vitro and in vivo studies, demonstrating its anti-SARS-CoV-2 activity [84]. Heat transfer tests have shown that PF-00835231 exhibited high affinity and specificity in binding to M^pro^ [84,131,132]. Moreover, the FRET protease activity test revealed its strong inhibitory effects on the Alpha (B1.1.7), Beta (B.1.351), Gamma (P.1), Delta (B.1.617.2), and Omicron (B.1.1.529) variants of SARS-CoV-2 in vitro [133,134]. PF-07321332 (Nirmatrelvir), derived from PF-07304814, is an oral M^pro^ inhibitor that exhibits high effectiveness. It is designed to optimize oral bioavailability and also possesses anti-inflammatory properties, inhibiting major proteases of α and β-coronaviruses, including SARS-CoV-1, HKU1, OC43, MERS, 229E, and NL63 [135,136]. Moreover, PF-07321332 has efficacy against emerging SARS-CoV-2 variants, such as Lambda (C.37), B.1.1.318, B.1.2, Beta (B.1.351), Omicron (B. 1.1.529), Zeta (P.2), and Delta (B.1.617.2), highlighting its broad effectiveness against SARS-CoV-2 throughout the pandemic [137,138]. Danoprevir, a repurposed non-covalent HCV proenzyme inhibitor, has shown positive results in terms of antiviral efficacy when administered orally in combination with ritonavir in patients with COVID-19. It has completed Phase 4 clinical studies [88,139]. Dexamethasone, a drug with significant anti-inflammatory effects, is currently in phase 4 clinical trials for the treatment of COVID-19 [88]. It exhibits high binding affinity primarily to glucocorticoid receptors and various cytokines (such as interleukin-6), and it has also shown potential as an M^pro^ inhibitor [140,141]. Additionally, polycycline, a drug known for its anti-inflammatory properties, is currently undergoing Phase IV clinical trials for COVID-19 treatment [142]. While polycycline has demonstrated activity against SARS-CoV-2 in vitro, its inhibitory effect on M^pro^ is supported by in silico studies [143,144]. Furthermore, among the drugs showing promising results and advancing to clinical trials, there are hepatitis C antiviral drugs such as telaprevir, narlaprevir, and boceprevir, which inhibit M^pro^. Additionally, drugs like remdesivir, ribavirin, suramin, and favipiravir inhibit RdRp and glycoside triphosphate (RTP) [145,146,147,148,149,150]. Overall, SARS-CoV-2 M^pro^ inhibitors present a promising approach for the treatment of coronavirus infections, either as single treatments or in combination with other antiviral agents [151].

## 6. Discussion

The COVID-19 global pandemic, caused by the novel coronavirus (SARS-CoV-2), has persisted for three years, resulting in significant and far-reaching impacts on people’s daily lives. Currently, the Omicron subfamily of the virus is still undergoing mutations, posing ongoing threats to human life and health. Therefore, the research and development of vaccines and antiviral drugs for SARS-CoV-2 remain of paramount importance. M^pro^, a critical cysteine protease in the coronavirus replication cycle, plays a vital role in cleaving 11 sites on the precursor polymers to release various non-structural proteins required for the virus’s life cycle. Consequently, the development of M^pro^ inhibitors that can block its proteolytic activity holds the potential to effectively impede virus replication and achieve antiviral effects. Currently, over 1000 M^pro^ structures have been stored in the PDB, and related studies have provided detailed descriptions of the binding characteristics and catalytic mechanism of M^pro^ with its substrates. This facilitates the development of antiviral drugs based on the structure of M^pro^, and numerous potentially effective inhibitors have been screened and validated. Moreover, the self-maturation mechanism of M^pro^ has been gradually elucidated, opening up promising avenues for the development of effective antiviral drugs targeting key steps in this process. In terms of clinical treatment, a significant development is the formal approval of Pfizer’s novel coronavirus infection treatment drug, Paxlovid, by the US FDA on 25 May 2023. Additionally, antiviral drugs targeting M^pro^, such as dexamethasone and doxycycline, have reached the final stages of clinical research, indicating their potential as treatment options. This study aims to provide a comprehensive summary of the structure and catalytic mechanism of the novel coronavirus M^pro^, along with the current primary methods employed in drug development targeting M^pro^. The objective is to offer systematic and valuable information for the development of antiviral drugs targeting M^pro^ and the treatment of COVID-19.

It is crucial to recognize that while M^pro^ may be relatively conserved among CoVs, it is still subject to variation. The evolutionary pressure exerted by the widespread use of antiviral drugs like nitrolimus/ritonavir can lead to the emergence of new viral variants with M^pro^ mutations, resulting in immune envision and drug resistance. Understanding the potential locations and specific mutations in M^pro^ that can lead to these effects is essential for guiding the development of new antiviral drugs that can effectively combat drug-resistant virus variants. However, there have been limited reports on this topic thus far. Therefore, conducting further in-depth research and exploration into the key potential mutations of M^pro^ is crucial in preparing for future infections caused by new virus variants. This analysis will also aid in the design of antiviral drugs with broad-spectrum activity against CoVs.

## Figures and Tables

**Table 1:** Inhibitors targeting the SARS-CoV-2 M^pro^ and their research and development status.

Drug Name	Mechanism	The Highest Research and Development Status of Drugs (Global)	Research Institution	Ref.
Leritrelvir (RAY1216)	RAY1216 has a drug-target residence time of 104 min. RAY1216 is covalently attached to the catalytic Cys145 through the α-ketoamide warhead. RAY1216 (0–1000 nM; 72 h) shows antiviral activities against SARS-CoV-2 wild-type ancestral strain and variants.	Approved for listing	Guangdong Raynovent Biotech Co., Ltd., Guangzhou, China	NCT05620160
Ensitrelvir Fumaric Acid (Ensitrelvir, S-217622)	Ensitrelvir (S-217622) is the first orally active non-covalent, non-peptidic SARS-CoV-2 main protease inhibitor (IC50 = 13 nM). In a cytopathic effect (cpe)-inhibition assay of SARS-CoV-2 infected VeroE6/TMPRSS2 cells, Ensitrelvir shows the EC50 values of approximately 0.4 μM for both wild-type virus and Alpha, Beta, Gamma, and Delta variants. EC50 values for SARS-CoV and MERS-CoV were 0.21 and 1.4 μM, respectively.	Approved for listing	Hokkaido University; Shionogi & Co., Ltd., Sapporo, Japan	[152,153,154,155]
QLS1128	As a potent M^pro^ inhibitor with SARS-CoV-2 antiviral activity.	Clinical Phase 3	Qilu Pharmaceutical Co., Ltd., Jinan, China	NCT05758519
STI-1558	As a potent M^pro^ inhibitor with SARS-CoV-2 antiviral activity.	Clinical Phase 3	Texas A&M University, Sorrento Therapeutics, Inc., San Diego, CA, USA	NCT05716425
Lufotrelvir (PF-07304814)	Lufotrelvir (PF-07304814), a phosphate proagent of PF-00835231, acts as a potent 3CLpro protease (M^pro^) inhibitor with SARS-CoV-2 antiviral activity. Lufotrelvir binds and inhibits SARS-CoV-2 M^pro^ activity with a Ki of 174 nM. Lufotrelvir is promising single antiviral agent that can be used for research on combination with other antivirals that target other critical stages of the coronavirus life cycle.	Clinical Phase 3	Pfizer Inc., New York, NY, USA	[136,155]
Bofutrelvir (FB-2001, FB 2001, DC402234)	Bofutrelvir (FB2001) is a SARS-CoV-2 main protease inhibitor with an IC_50_ value of 53 nM and an EC_50_ value of 0.53 μM. Bofutrelvir exhibits potent antiviral efficacy against several current SARS-CoV-2 variants with EC_50_ values of 0.26–0.42 μM. Bofutrelvir has an additive antiviral effect when combined with Remdesivir (HY-104077)	Clinical Phase 2/3	Shanghai Institute of Materia Medica; Frontier Biotechnologies, Nanjing, China	[156,157]
Nirmatrelvir (PF07321332)	Nirmatrelvir (PF-07321332) is a potent and orally active SARS-CoV M^pro^ inhibitor. Nirmatrelvir (PF-07321332) targets to the SARS-CoV-2 virus and can be used for COVID-19 research	Clinical Phase 2/3	Pfizer Inc., New York, NY, USA; University of California	[136]
GST-HG171	As a potent M^pro^ inhibitor with SARS-CoV-2 antiviral activity.	Clinical Phase 2/3	Fujian Akeylink Biotechnology Co., Ltd., Ningde, China	NCT05656443
WPV01(Mprosevir)	As a potent M^pro^ inhibitor with SARS-CoV-2 antiviral activity.	Clinical Phase 2	Westlake University	[158]
EDP-235 (Direct acting antivirals—Enanta Pharmaceutical)	As a potent M^pro^ inhibitor with SARS-CoV-2 antiviral activity.	Clinical Phase 2	Enanta Pharmaceuticals, Inc., Watertown, MA, USA	[159]
Tollovir (NLC-V-01)	As a potent M^pro^ inhibitor with SARS-CoV-2 antiviral activity.	Clinical Phase 2	Todos Medical Ltd., Tel Aviv, Israel	NCT05226767
PF-07817883	As a potent M^pro^ inhibitor with SARS-CoV-2 antiviral activity.	Clinical Phase 2	Pfizer Inc.	NCT05799495
ABBV-903	As a potent M^pro^ inhibitor with SARS-CoV-2 antiviral activity.	Clinical Phase 1	AbbVie, Inc., North Chicago, IL, USA	NCT05691699
ALG-097558	As a potent M^pro^ inhibitor with SARS-CoV-2 antiviral activity.	Clinical Phase 1	Aligos Therapeutics, Inc., San Francisco, CA, USA; Rega Institute for Medical Research, Leuven, Belgium	NCT05840952
EDDC-2214	As a potent M^pro^ inhibitor with SARS-CoV-2 antiviral activity.	Clinical Phase 1	Experimental Drug Development Centre, Singapore	[160]
SAL0133	As a potent M^pro^ inhibitor with SARS-CoV-2 antiviral activity.	Clinical Phase 1	Shenzhen Salubris Pharmaceuticals Co., Ltd., Shenzhen, China	[160]
GS-221	As a potent M^pro^ inhibitor with SARS-CoV-2 antiviral activity.	Clinical Phase 1	Grand Pharmaceutical Group Ltd., Wuhan, China	NCT03013998
SYH-2055	Acts on the main protease of SARS-CoV-2 andinhibits the cleavage of viral precursor protein, thereby blocking viral replication and playing the role of anti-SARS-CoV-2.	Clinical Phase 1	CSPC Pharmaceutical Group Ltd., Shijiazhuang, China	[160]
GS-00202	As a potent M^pro^ inhibitor with SARS-CoV-2 antiviral activity.	Clinical Phase 1	Anovent Pharmaceuticals Co. Ltd., Shanghai, China	[160]
ASC11	As a potent M^pro^ inhibitor with SARS-CoV-2 antiviral activity.	Clinical Phase 1	Ascletis Pharma, Inc., Hangzhou, China	NCT05718518
ISM-3312	As a potent M^pro^ inhibitor with SARS-CoV-2 antiviral activity.	Clinical Phase 1	InSilico Medicine (Shanghai) Ltd., Shanghai, China	[160]
CDI-988	As a potent M^pro^ inhibitor with SARS-CoV-2 antiviral activity.	Clinical application	Cocrystal Pharma, Inc., Bothell, WA, USA	[160]
SBFM-PL4	As a potent M^pro^ inhibitor with SARS-CoV-2 antiviral activity.	Preclinical	Sunshine Biopharma, Inc., Fort Lauderdale, FL, USA	[160]
GRL-0820	As a potent M^pro^ inhibitor with SARS-CoV-2 antiviral activity.	Preclinical	Sunshine Biopharma, Inc.	[161]
CDI-783	As a potent M^pro^ inhibitor with SARS-CoV-2 antiviral activity.	Preclinical	Cocrystal Pharma, Inc.	[162,163]
CN-2021	As a potent M^pro^ inhibitor with SARS-CoV-2 antiviral activity.	Preclinical	Andikang (Wuxi) Biotechnology Co., Ltd., Wuxi, China	NCT05208853
LHP803(COR-803)	As a potent M^pro^ inhibitor with SARS-CoV-2 antiviral activity.	Preclinical	Quince Therapeutics, Inc., San Francisco, CA, USA	[160]
ISM036-076	As a potent M^pro^ inhibitor with SARS-CoV-2 antiviral activity.	Preclinical	InSilico Medicine, Inc.	[160]
3CLproPROTACs (Shaanxi Pa)	As a potent M^pro^ inhibitor with SARS-CoV-2 antiviral activity.	Preclinical	Shaanxi University of Science and Technology; Shaanxi Panlong Pharmaceutical Co., Ltd., Xi’an, China	[160]
CVD-0013943	As a potent M^pro^ inhibitor with SARS-CoV-2 antiviral activity.	Preclinical	Shaanxi Panlong Pharmaceutical Co., Ltd.	[160]
M^pro^13b-K (SARS-CoV-2 M^pro^ α-ketoamide inhibitor 13b-K)	As a potent M^pro^ inhibitor with SARS-CoV-2 antiviral activity.	Preclinical	Universität zu Lübeck; Tocris Bioscience, Bristol, UK	[160]
Sangivamycin (TNX350)	Sangivamycin (NSC 65346), a nucleoside analog, is a potent inhibitor of protein kinase C (PKC) with an K_i_ of 10 μM. Sangivamycin has potent antiproliferative activity against a variety of human cancers.	Preclinical	Tonix Pharmaceuticals Holding, Chatham, NJ, USA	[164,165]
YK-1007	As a potent M^pro^ inhibitor with SARS-CoV-2 antiviral activity.	Preclinical	Syntron (Jiangsu) Inc., Nanjing, China	[160]
VV993 (Anti-SARS-CoV-2 3CLpro inhibitor)	As a potent M^pro^ inhibitor with SARS-CoV-2 antiviral activity.	Preclinical	Shanghai Institute of Materia Medica Chinese Academy of Sciences, Shanghai, China	[160]
MI-30	As a potent M^pro^ inhibitor with SARS-CoV-2 antiviral activity.	Preclinical	Sichuan University	[160]
CDI-45205	As a potent M^pro^ inhibitor with SARS-CoV-2 antiviral activity.	Preclinical	Kansas State University Research Foundation	[136]
INSCoV-614(1B)	As a potent M^pro^ inhibitor with SARS-CoV-2 antiviral activity.	Preclinical	InSilico Medicine (Shanghai) Ltd.	[160]
AB-343(Arbutus Biopharma	As a potent M^pro^ inhibitor with SARS-CoV-2 antiviral activity.	Preclinical	Arbutus Biopharma Corp., Warminster, PA, USA	[160]
ENU-200(SARS-CoV-2 S glycoprotein/protease protein inhibitor)	It is a SARS-Cov-2 M^pro^ inhibitor and S glycoprotein inhibitor.	Preclinical	Ennaid Therapeutics LLC, Charlotte, NC, USA	[160]
CDI-873	As a potent M^pro^ inhibitor with SARS-CoV-2 antiviral activity.	Preclinical	Cocrystal Pharma, Inc.	[160]
CR-0305	As a potent M^pro^ inhibitor with SARS-CoV-2 antiviral activity.	Preclinical	Coeurative, Inc., Roanoke, VA, USA	[160]
GDI-4405	As a potent M^pro^ inhibitor with SARS-CoV-2 antiviral activity.	Preclinical	Global Health Drug Discovery Institute, Beijing, China	[160]
COR-817	As a potent M^pro^ inhibitor with SARS-CoV-2 antiviral activity.	Preclinical	Quince Therapeutics, Inc.	[160]

## Data Availability

All data included in this study are available.
